# Reproducibility: reliability and agreement parameters of the Revised Short McGill Pain Questionnaire Version-2 for use in patients with musculoskeletal shoulder pain

**DOI:** 10.1186/s12955-020-01617-4

**Published:** 2020-11-11

**Authors:** Samuel U. Jumbo, Joy C. MacDermid, Tara L. Packham, George S. Athwal, Kenneth J. Faber

**Affiliations:** 1 Faculty of Health and Rehabilitation Sciences, Elborn College, Western University, London, ON Canada; 2 School of Rehabilitation Science, McMaster University, 1400 Main Street West, Hamilton, ON Canada; 3 Roth McFarlane Hand and Upper Limb Centre, St. Joseph’s Hospital, London, ON N6A 4L6 Canada

**Keywords:** Reproducibility, Reliability, Agreement, McGill pain questionnaire, Shoulder pain, Musculoskeletal conditions, Patient-reported outcomes, Psychometric properties

## Abstract

**Background:**

The Revised Short McGill Pain Questionnaire Version-2 (SF-MPQ-2) is a multidimensional outcome measure designed to capture, evaluate and discriminate pain from neuropathic and non-neuropathic sources. A recent systematic review found insufficient psychometric data with respect to musculoskeletal (MSK) health conditions. This study aimed to describe the reproducibility (test–retest reliability and agreement) and internal consistency of the SF-MPQ-2 for use among patients with musculoskeletal shoulder pain.

**Methods:**

Eligible patients with shoulder pain from MSK sources completed the SF-MPQ-2: at baseline (n = 195), and a subset did so again after 3–7 days (n = 48), if their response to the Global Rating of Change (GROC) scale remained unchanged. Cronbach alpha (α) and intraclass correlation coefficient (ICC_2,1_), and their related 95% CI were calculated. Standard error of measurement (SEM), group and individual minimal detectable change (MDC90), and Bland–Altman (BA) plots were used to assess agreement.

**Results:**

Cronbach α ranged from 0.83 to 0.95 suggesting very satisfactory internal consistency across the SF-MPQ-2 domains. Excellent ICC_2,1_ scores were found in support of the total scale (0.95) and continuous subscale (0.92) scores; the remaining subscales displayed good ICC_2,1_ scores (0.78–0.88). Bland–Altman analysis revealed no systematic bias between the test and retest scores (mean difference = 0.13–0.19). While the best agreement coefficients were seen on the total scale (SEM = 0.5; MDC_90individual_ = 1.2 and MDC_90group_ = 0.3), they were acceptable for the SF-MPQ-2 subscales (SEM: range 0.7–1; MDC_90individual_: range 1.7–2.3; MDC_90group_: range 0.4–0.5).

**Conclusion:**

Good reproducibility supports the SF-MPQ-2 domains for augmented or independent use in MSK-related shoulder pain assessment, with the total scale displaying the best reproducibility coefficients. Additional research on the validity and responsiveness of the SF-MPQ-2 is still required in this population.

## Background

Shoulder disorders are among the three leading causes of musculoskeletal (MSK) pain, third only to neck pain and low back pain [1, 2]. The prevalence of shoulder disorders increases with aging [3, 4]. Shoulder disorders are associated with substantial consequences for the socioeconomic wellbeing of the patient and society; studies have linked workers’ absenteeism, job loss, and poor health-related quality of life (HRQoL) to symptoms associated with shoulder disorders [3, 5–8].

Pain assessment in clinical practice and research often places emphasis on monitoring pain intensity, even though pain is known to be multidimensional and experienced uniquely by individuals [9]. Patients perceive pain across six diverse dimensions: physiologic, sensory, affective, cognitive, behavioral and socio-cultural [9, 10]. The comprehensive assessment and monitoring of these dimensions should improve patient care [11]. A multidimensional pain assessment tool that provides a holistic assessment of pain has been recommended by experts [12–14] for use in upper extremity conditions, including shoulder disorders.

The Revised Short McGill Pain Questionnaire Version-2 (SF-MPQ-2) is an example of a general use multidimensional pain tool that comprehensively examines the sensory and affective dimensions of pain. Dworkin et al. [15] created the SF-MPQ-2 by adding seven new items that explicitly examines neuropathic and non-neuropathic pain characteristics to the original 15-item Short McGill Pain Questionnaire (SF-MPQ). They also replaced the previous 4-point descriptive rating scale with a 10-item numerical rating scale to enhance its responsiveness [15]. Since then, multiple studies have utilized the SF-MPQ-2 as a primary outcome for pain assessment in clinical trials; its measurement properties have been examined in different populations including cancer pain [16], surgical pain [17], visceral pain [18], and neuropathic pain [19]. Among MSK conditions, studies have reported measurement evidence for patients with complex regional pain syndrome [20], back pain [21], knee osteoarthritis (OA) [22], and mixed MSK populations [23, 24]. Although the SF-MPQ-2 is becoming increasingly popular, our recent review [25–27] reported on evidence with design flaws including inadequate description of Intraclass correlation coefficient (ICC) models, insufficient justification of retest interval, and a lack of attention to absolute reliability parameters.

In the absence of such evidence, the primary purpose of this study was to investigate the reproducibility (test–retest reliability and agreement) and internal consistency of the Revised Short McGill Pain Questionnaire Version-2 (SF-MPQ-2) among persons with MSK-related shoulder disorders.

## Methods

This study was based on a cross-sectional study of internal consistency and test–retest reliability. The SF-MPQ-2 questionnaire was administered to examine reproducibility (test–retest reliability and agreement) and internal consistency at two time points: at baseline and after 3–7 days (when patients would, for the most part, be stable) [28, 29]. The participants were recruited from the Roth-McFarlane Hand and Upper Limb Centre (HULC), London, Ontario, Canada during a period of 6-months (June to November 2018). Ethics approval for a clinical database of routine outcome measures from which this data were extracted was approved by the University of Western Ontario Research Ethics Board (REB# 4986).

### Patients

Adults proficient in English, above 18 years of age, that experienced pain from one or more shoulder conditions of known MSK source (for example: rotator cuff tear or tendinopathy, adhesive capsulitis, glenohumeral anterior instability, and superior labral anterior–posterior (SLAP) lesions) were included. Potential participants were excluded if they had: (1) an unstable cardiorespiratory condition; (2) any history of problems relating with the central nervous system e.g. hemiplegia; (3) pain resulting from neoplastic or infectious or vascular disorders or referred from internal organs; (4) any neuropathic pain symptoms resulting from thoracic outlet syndrome, carpal tunnel syndrome or any peripheral nerve entrapment, or (5) did not provide consent.

### Procedure

Assessors (SJ and HULC research assistants) identified eligible participants by reviewing the outpatient appointment list of patients scheduled for a clinical visit with two shoulder surgeons (KF and GA), a day prior. Potential participants were then contacted on the day of their clinical appointment and screened to ensure all criteria were satisfied; they were provided with an explanation of the objectives of the study before a questionnaire booklet containing the SF-MPQ-2 and Global Rating of Pain Scale (GROC) was administered. Each participant was verbally instructed to carefully read and circle the response that described their pain experience. In cases where participants had difficulty with selecting an answer, they were told to choose the answer that comes closest to describing their pain symptoms. If help was needed with understanding any words or phrases, or with marking their responses, the assessors assisted. The participants were instructed to complete all items in the questionnaire. Participants were permitted to withdraw from the study for any reason at any time. For the second test occasion, a subset of the participants (102 in total) that verbally confirmed being in unchanged/stable pain in the past 7-days were conveniently sampled to self-complete the SF-MPQ-2 and GROC at home within 3–7 days, if their pain remained unchanged (i.e. if they could confirm that the threshold of their perceived pain for their shoulder disorder had not changed in the past week). The GROC scale was administered, intentionally, on both test occasions solely to serve as an objective means of comparing participants test and retest responses thus ensuring that only participants in stable/unchanged pain conditions were included in our analysis of reproducibility (test–retest reliability and agreement). Demographic information including age, hand dominance, primary cause of shoulder pain and sex were recorded.

### Outcome measure

The Revised Short McGill Pain Questionnaire Version-2 (SF-MPQ-2) contains 22-items/pain descriptors and 4 subscales/domains that examine pain intensity and quality as follows: (1) continuous pain (throbbing, cramping, gnawing, aching, heavy, and tender pain); (2) intermittent pain (shooting, stabbing, sharp pain, splitting pain, electric-shock, and piercing pain); (3) neuropathic pain (hot-burning, cold-freezing, pain caused by light touch, itching, tingling or pins and needles, and numbness pain), and (4) affective pain (tiring-exhausting, sickening, fearful, and punishing-cruel). All the items are bounded on a zero (none) to 10 (worst possible) numerical rating scale. The mean of the 22-items yields the SF-MPQ-2 total score, while the mean of the items that comprise each of four-subscales yields the summary score for the subscale [15, 21]. Higher subscale or total scores suggest greater pain symptoms/experience, and more than 2 missing values renders patients’ response to the questionnaire invalid [21]. The SF-MPQ-2 uses a recall period of 7-days, instructing the person to base their rating on their symptoms in the past week [15].

### Statistical analyses

The SF-MPQ-2 total and subscale scores were considered as interval variables. Data quality and screening, including the percentage of missing data, outliers, and presence of floor/ceiling effects was performed. Respondents with two or more missing items were excluded, in line with the developers’ instructions [21]. Continuous variables were descriptively summarized using means and standard deviations while percentages were used to report categorical variables. The data were then examined for normality with histograms, and the Shapiro–Wilk test. All statistical analyses were completed with Microsoft Excel Version 2013 and SPSS statistic for Windows™, Version 25.0. (Armonk, NY: IBM Corp, Released 2017).

#### Floor/ceiling effects

Floor/ceiling effects for the SF-MPQ-2 were assessed by identifying the number of participants with the absolute lowest (0-points = floor) and highest (10-points = ceiling) scores on the total and subscales. Floor/ceiling effects occurring at the magnitude of 15% were considered substantial [30].

##### Hypothesis:

We expected substantial floor effects on the neuropathic and affective subscales of the SF-MPQ-2 because they evaluate pain dimensions that are relatively uncommon in orthopaedic shoulder disorders.

#### Cross sectional reliability (internal consistency)

Internal consistency, the degree of item inter-relatedness/equivalence in a Patient-Reported Outcome Measure (PROM) [30–32], was assessed with Cronbach alpha (α) and associated 95% confidence intervals. An α ≥ 0.7 is a commonly accepted standard for internal consistency reliability. However, redundancy is suggested at α > 0.95 [30, 32, 33].

##### Hypothesis:

We expected the SF-MPQ-2 to be internally consistent with Cronbach α at 0.8 or above for its subscale scores, and 0.9 or above for its total scores as previously reported in the literature [22, 24].

#### Relative reliability (test–retest reliability)

The intraclass correlation coefficient (ICC_2,1_) was used to assess the retest reliability of the SF-MPQ-2 total and subscales [34]. ICC_2, 1_ with 95% confidence intervals (CI) were computed using the two-way mixed and absolute agreement model, that assumes the patients were randomly selected but the occasions were fixed choices [35]. We chose an ICC_2,1_ absolute agreement over a consistency model because it captures elements of systematic bias and is preferred for computing an absolute reliability indicator. ICC_2,1_ values for the SF-MPQ-2 total and subscale scores were considered Negative ≤ 0.49, Doubtful 0.50–0.69, Good 0.70–0.89, and Excellent 0.90–1.00 [36].

##### Hypothesis:

We expected good ICC_2,1_ scores for group level analysis at ≥ 0.80 for the total scale and ≥ 0.70 for the subscale scores as previously reported in the literature [22, 24].

#### Agreement properties (standard error of measurement [SEM] and minimal detectable change [MDC])

Standard error of measurement (SEM) is defined as the standard deviation of errors of measurement associated with particular test takers’ scores [37]. Table 1 explains the five equations used for agreement analysis. To define SEM_agreement_ for the SF-MPQ-2 total and subscales scores, the pooled standard deviation calculated from participants’ mean responses to the SF-MPQ-2 domains on both test and retest using Eq. 1 [37, 38] and the respective non-transformed ICC_2,1_ for the SF-MPQ-2 domain under evaluation was keyed into Eq. 2 [37–39] (Table 1). Further, the proportion of the resulting SEM per domain to the total score of the scale was calculated to yield the SEM percentage or SEM%, as previously used [39–41] and interpreted as follows: ≤ 5% = very good; > 5–≤ 10% = good; > 10–< 20% = doubtful; and values above 20% = negative [39].

**Table 1:** Summary of equations used in agreement analysis

Equation	Formula	Purpose
1	SD_pooled_ = (SD_test_ + SD_retest_)/2	For estimating pooled standard deviation (SD_pooled_) from the test and retest scores. The SD_pooled_ is among the indices required for SEM_agreement_ estimation
2	SEM_agreement_ = Standard Deviation_pooled_ ×$\sqrt{1}-\mathrm{ICC}$_2,1_	For estimating SEM_agreement_, which is important for the MDC90_individual_ estimation
3	MDC_90individual_ = 1.64 × √2 × SEM_agreement_	For determining the point estimate of MDC_90individual_, which is required for estimating the confidence interval range and the MDC_90group_ scores per subscale of the SF-MPQ-2
4	95% CI for MDC_90individual_ = d ± MDC_90individual_	For computing the 90% confidence interval range for the MDC_90individual_ score obtained for each subscale of SF-MPQ-2
5	MDC_90group_ = MDC_90individual_ /√n × 1.64	For estimating the MDC_90 group_ score for the entire population

*SEM*_*agreement*_ standard error of measurement (agreement), *SD*_*test*_ standard deviation of test scores, *SD*_*retest*_ standard deviation of retest scores, *SD*_*pooled*_ pooled standard deviation, *n* sample size, *CI* confidence interval, *MDC*_*90individual*_ individual level minimal detectable change at 90% CI, *MDC*_*90group*_ group level minimal detectable change at 90% CI, *d* mean difference, *ICC*_*2,1*_ intraclass correlation coefficient

The minimal detectable change (MDC) or repeatability coefficient describes the minimum amount of change that must occur on a score to be confident that true/real change (that may or may not be clinically significant) has occurred without error after two repeated measures, within the period of the test–retest [42]. For this study, a 90% confidence interval was estimated for the Minimal Detectable Change (MDC_90_). Like the SEM, it is also expressed in the unit of the measure and may be computed at an individual level (MDC_90individual_) or for a group (MDC_90group_) [29]. We estimated MDC_90individual_ for the total and subscale scores of the SF-MPQ-2 by entering each scale’s SEM_agreement_ into Eq. 3 (Table 1) assuming the data was normally distributed and free of systematic error. The MDC_90individual_ confidence interval was then computed from the mean differences (d) of each subscale using Eq. 4 (Table 1) [29, 40, 43].

To determine the group level minimal detectable change (MDC_90group_), which is useful for determining if changes have occurred in an entire population, Eq. 5 (Table 1), the formula proposed by de Vet et al. [30, 44] was employed. The proportion of the resulting MDC coefficient per SF-MPQ-2 domain to the total score of the scale was computed to yield the MDC percent score (MDC%) and interpreted as follows: ≤ 5% = very good; > 5–≤ 10% = good; > 10–< 20% = doubtful; and values above 20% = negative [39, 40].

#### Bland–Altman Plots (BA Plots)

The Bland–Altman (BA) method was used to visually examine the agreement between the test and retest scores [45, 46]. Scatter plots were created to demonstrate the differences between the total and subscale scores obtained at time one and time two of the test–retest interval against their mean score for the two time points [45–48]. We then calculated the mean difference between the two measurement intervals (the ‘bias’) and the 95% limits of agreement (LoA) using: LoA = mean difference (d) ± 1.96 SD of the mean differences. The BA plots were used to visually judge the 95% limits of agreement to determine how well the scores from repeated measurements agreed: narrower LoAs suggested better agreement at the individual level [29, 47, 49]. Agreement at the group level was determined by how close the bias (mean difference) was to zero. Also, the distribution of scatter points on the BA plots were visually scrutinized for evidence of variability or heteroscedasticity, where the absence of a linear relationship between test–retest mean differences and their mean scores, per subscale, suggest the absence of systematic bias [44–48, 50]. Linear regression models were used to explore the presence of systematic bias. For each domain of the SF-MPQ-2, mean scores and differences in mean scores were modelled as the independent and dependent variables, respectively. The potential for systematic bias was appraised by checking if the prediction of the differences in the mean scores was statistically significant [47, 51]. Finally, outliers that presented beyond the upper and lower boundaries of the LoA were noted and explored [29, 52].

## Results

**Fig. 1:**
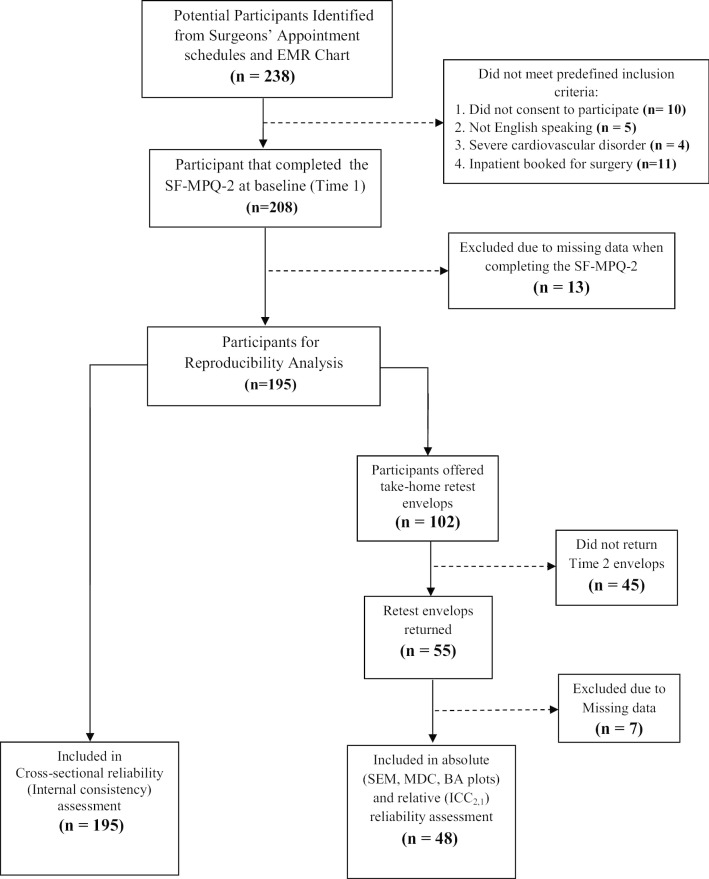
Flow chart of progress through the phases of screening, recruitment, test, retest and data analysis

Figure 1 below summarizes the flow of participants through the different phases of the study. Of the 238 eligible patients identified from the review of the surgeons’ scheduled appointment list, 195 consenting adults satisfied the inclusion criteria and provided complete data that were considered in our analysis of cross-sectional reliability. For the analysis of test–retest reliability and agreement, of the 102 participants that agreed to participate in the second test occasion, only 48 out of 55 stable subjects provided a complete response to SF-MPQ-2 in a mean of 4 days following the index test.

Table 2 summarizes the characteristic and demographic distribution of the baseline population. The study population was equally comprised of males and females, with a mean age of 62 years, with different shoulder disorders of various MSK pathologies including rotator cuff injuries, humeral fracture and glenohumeral joint arthroplasty.

**Table 2:** Patient baseline characteristics (N = 195)

Variables	N/%
Age in years (mean + SD)	(62 + 17) 195/100%
*Shoulder disorders*	
Glenohumeral joint arthroplasty	39/20%
Humeral and others fractures (i.e. clavicular, costal, scapular)	23/12%
Rotator cuff pathologies	48/25%
Dislocation	12/6%
Osteoarthritis	18/9%
Impingement/bursitis	15/8%
Other (MSK pain-related/non-specific)	40/21%
*Affected shoulder*	
Right	111/56%
Left	71/36%
Both	13/6%
*Sex*	
Males	103/53%
Females	92/47%

*N* number of patients, *SD* standard deviation

Both the graphical and statistical tests of normality revealed the dataset was skewed/abnormal. To address the assumption of normality for further analysis, a square root calculation was used to transform the data. A closer look at the reliability coefficients obtained using the transformed and untransformed data revealed only a small difference in scores (see Table 3 for results). Parametric statistics were used in our analysis because the sample size was greater than 30 participants (based on the central limit theorem). Despite that, we still examined for differences in reproducibility coefficients obtained using the transformed and non-transformed ICC scores.

**Table 3:** Floor and ceiling effects for test–retest scores of the SF MPQ-2 total and subscale scores (N = 48)

Variables	Test		Retest	
	Floor	Ceiling	Floor	Ceiling
SF-MPQ-2 Continuous	7/48 = 15%	0 /48 = 0%	4/48 = 8%	1/48 = 2%
SF-MPQ-2 Intermittent	11/48 = 23%	0/48 = 0%	15/48 = 31%	0/48 = 0%
SF-MPQ-2 Affective	19/48 = 40%	1/48 = 2%	20/48 = 42%	0/48 = 0%
SF-MPQ-2 Neuropathic	14/48 = 29%	0/48 = 0%	11/48 = 23%	0/48 = 0%
SF-MPQ-2 Total	3/48 = 6%	0/48 = 0%	4/48 = 8%	0/48 = 0%

*SF-MPQ-2* Revised Short McGill Pain Questionnaire Version-2, *%* proportion in percentages

### Floor and ceiling effects

The presence of floor/ceiling effect may suggest an outcome measure is not responsive to detecting improvement (ceiling effect) even though a decline in status can be captured, and vice versa for floor effects [21]. The number of patients who obtained the absolute maximum (Ten, 10) and minimal (zero, 0) scores on the SF-MPQ-2 total and subscales are summarized in Table 3. The greatest level of floor effect was observed on the affective subscale at both periods of the test–retest. Substantial floor effects were also noted on the neuropathic and intermittent subscales. None of the SF-MPQ-2 indices had remarkable ceiling effects.

### Internal consistency (cross-sectional reliability)

Table 4 summarizes the results obtained for cross sectional reliability. The SF-MPQ-2 displayed excellent internal consistency with robust α coefficients within a range that suggest the absence of redundancy: α coefficients for the total subscale peaked at 0.95 as posited, while that for the subscales fluctuated between 0.83 and 0.86 points. Inter-item correlations were satisfactory, ranging from 0.23–0.53 across the scales.

**Table 4:** Cross-sectional reliability of the SF-MPQ-2 total and subscale scores (N = 195)

Variables	Internal consistency (N = 195)	
	Cronbach alpha (95% CI)	Inter-item correlation
SF-MPQ-2 Continuous	0.87 (0.84–0.90)	0.43–0.67
SF-MPQ-2 Intermittent	0.87 (0.84–0.90)	0.42–0.77
SF-MPQ-2 Neuropathic	0.85 (0.81–0.88)	0.32–0.81
SF-MPQ-2 Affective	0.83 (0.79–0.87)	0.44–0.78
SF-MPQ-2 Total	0.95 (0.94–0.96)	0.21–0.78

*SF-MPQ-2* Revised Short McGill Pain Questionnaire Version-2, *CI* confidence interval

### Agreement properties (absolute test–retest reliability)

Table 5 summarizes the agreement parameters supporting the SF-MPQ-2 domains. The total scale SEM_agreement_ was very low (0.51points) and approximately 5% of the total score of the scale, which is ‘very good’ according to our criteria. Individual subscale SEM_agreement_ ranged from 0.73 to 0.99 (approximately ≤ 10% of the total score), which is also ‘good’ according to our criteria. At the individual level, acceptable scores within 1.19–2.29 points were seen in support of minimal detectable change (MDC) at a 90% confidence level. Of all the SF-MPQ-2 domains, the total scale had the lowest MDC score at 1.20 points (i.e. 12%) while the intermittent subscale had the most substantial MDC scores at 2.29 points (i.e. 23%). For Group MDC_90_, estimates were acceptable and expectedly lower than those obtained for MDC_90individual_; the results fluctuated within 0.28 (total) to 0.54 (intermittent) points across the SF-MPQ-2 domains (Table 5).

**Table 5:** Agreement parameters (absolute reliability) of the SF-MPQ-2 total and subscale scores (N = 48)

Variables	SEM_agreement_	SEM (%)	MDC_90individual_ (95% CI)	MDC (%)	MDC_90group_
SF-MPQ-2 Continuous	0.8	8	1.8 (− 1.6 to 2.0)	18	0.4
SF-MPQ-2 Neuropathic	0.8	8	1.8 (− 1.7 to 1.9)	18	0.4
SF-MPQ-2 Intermittent	1.0	10	2.3 (− 2.1 to 2.4)	23	0.5
SF-MPQ-2 Affective	0.7	7	1.7 (− 1.5 to 1.8)	17	0.4
SF-MPQ-2 Total	0.5	5	1.2 (− 1.0 to 1.4)	12	0.3

*SF-MPQ-2* Revised Short McGill Pain Questionnaire Version-2, *CI* confidence interval, *SEM* standard error measurement, *MDC* minimal detectable change

*SEM (%) and MDC (%)* is expressed as the proportion of the obtained SEM_agreement_ or MDC_90individual_ of domain represented on the SF-MPQ-2 to the total score of the scale (i.e. 10 points)

### Relative test–retest reliability

The test–retest reliability of the SF-MPQ-2 domains was rated “Good” to “Excellent” (Table 6). Our results for ICC_2,1_ were based on an analysis conducted with the non-transformed data, as they did not differ from that obtained with transformed data. ICC_2,1_ scores were highest on the continuous and total subscales and rated excellent according to our criteria. The neuropathic, affective and intermittent subscales displayed good ICC_2,1_ coefficients (Table 6) in support of relative reliability.

**Table 6:** Relative reliability of the SF-MPQ-2 total and subscale Scores (N = 48)

Variables	Test–retest reliability						
	Test Mean (SD)	Test Mean (SD)	d (SD)	95% CI of d	95% LoA	Single measure ICC_2,1_ (95% CI)	
						Transformed data	Non-transformed data
SF-MPQ-2 Continuous	2.8 (2.6)	2.7 (2.6)	0.19 (1.12)	− 0.14 to 0.51	− 2.01, 2.38	^a^0.90 (0.83–0.94)	^a^0.91 (0.84–0.95)
SF-MPQ-2 Intermittent	2.1 (2.3)	2.0 (2.4)	0.15 (1.39)	− 0.24 to 0.54	− 2.58, 2.88	^a^0.82 (0.71–0.90)	^a^0.82 (0.71–0.90)
SF-MPQ-2 Neuropathic	1.5 (1.6)	1.3 (1.7)	0.13 (1.10)	− 0.19 to 0.45	− 2.02, 2.28	^a^0.78 (0.64–0.87)	^a^0.78 (0.64–0.87)
SF-MPQ-2 Affective	1.5 (1.9)	1.3 (2.0)	0.15 (1.01)	− 0.14 to 0.45	− 1.83, 2.14	^a^0.85 (0.75–0.92)	^a^0.87 (0.78–0.92)
SF-MPQ-2 Total	2.0 (1.9)	1.9 (2.0)	0.15 (0.73)	− 0.06 to 0.37	− 1.29, 1.59	^a^0.92 (0.86–0.96)	^a^0.93 (0.87–0.96)

*SF-MPQ-2* Revised Short-form McGill Pain Questionnaire Version-2, *d* mean difference (test–retest), *SD* standard deviation, *CI* confidence interval, *LoA* limits of agreement, *ICC* intraclass correlation coefficient

^a^All correlation coefficient (r) were statistically significant at *p* < 0.001 (2-tailed)

### Bland–Altman (BA) analysis/plots

**Fig. 2:**
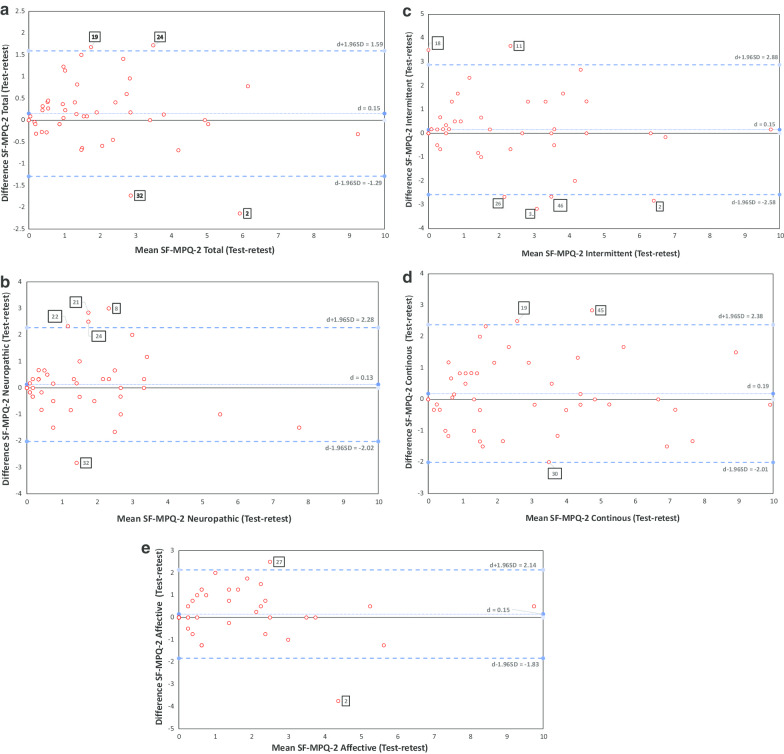
**a**–**e** The Bland–Altman Limits of Agreement (LoA) plots between the test and retest scores of the SF-MPQ-2 Total (**a**), Neuropathic (**b**), Intermittent (**c**), Continuous (**d**) and Affective (**e**) subscale scores (n = 48). The difference between test–retest scores is plotted against the mean of test and retest scores for the respective SF-MPQ-2 total and subscales depicted. On each plot, the central blue line represents the mean of intra individual differences (**d**); the upper and lower horizontal broken lines represent the 95% LoA. The 95% LoA shows that 95% of the intra individual differences are within ± 1.96 SD of the mean difference (**d**). The outlier noted in each BA plot is numbered, according to participant #RS I.D., and presented in accordance with the SF-MPQ-2 subscale or total scores in which they were noted

The results of our Bland–Altman analysis are presented in Table 6. The Bland–Altman plots superimposed with the LoA and mean difference (bias) scores for each domain of the SF-MPQ-2 are graphically illustrated (Fig. 2a–e). All of the SF-MPQ-2 domains displayed acceptable LoA at a 95% confidence level with the highest distance ranging 5 points (intermittent subscale). The total scale score displayed the narrowest LoA (range = 3 points), with the remaining subscales within satisfactory limits. Mean difference scores (bias) were very acceptable for all the SF-MPQ-2 domains (0.15–0.19 points).

Visual inspection of scatter points on the BA plots for each domain of the SF-MPQ-2 revealed that the magnitude of the mean differences against the mean scores were uniformly distributed from the zero point and most scatter points were within the 95% LoA with the exception of a few outliers. This supports the absence of systematic bias and suggest a good level of agreement among test–retest scores. Furthermore, for each of the SF-MPQ-2 domains, there was no evidence of the mean difference scores predicting the mean average after our regression model analysis. These findings suggest that systematic bias is unlikely and confirms good level of agreement between the test–retest scores (Table 6).

The few outliers noted were explored. First, we determined if they were erroneous responses in entry by rechecking hard copies but, indeed, they were ‘interesting’ outliers [53] and labelled according to their #RS on each BA plot. The greatest number of interesting outliers presented on the intermittent (n = 6, 12%) and neuropathic (n = 4, 10%) subscales. The least number of outliers were seen on the affective subscale (n = 2, 4%). In general, however, the presence of these outliers did not indicate the presence or absence of bias [53].

## Discussion

This study provides reproducibility evidence that supports the use of the SF-MPQ-2 in multidimensional pain assessment of people with MSK shoulder pain. The SF-MPQ-2 displayed good to excellent coefficients in support of its relative reliability and absolute reliability properties. The limits of agreement for the subscales and total scores were very satisfactory.

The substantial floor effect observed on the neuropathic, intermittent and affective subscales can be attributed to the robust discriminative properties of the SF-MPQ-2 subscales and to the lower prevalence of these problems in our study population. Conceptually, the SF-MPQ-2 was expanded to provide a single tool that can classify pain between neuropathic and non-neuropathic sources [15, 21]. As outcome measures can be evaluative or discriminative, combining both purposes within an outcome measure is likely to result in these types of statistical issues. For instance, participants with pain emerging from neuropathic sources will be more inclined to respond adequately to the neuropathic subscale, thereby reducing the likelihood of floor effects. This has been observed with the use of the SF-MPQ-2 among complex regional pain syndrome (CPRS) patients [20]. This implies that floor effects on the SF-MPQ-2 domains may not always represent redundancy, but rather, may suggest that an item does not describe the patient’s pain experience [25].

Cross sectional reliability was established for the SF-MPQ-2 total and subscale scores with satisfactory coefficients supporting internal consistency that are similar to previous estimates among mixed-MSK[23] (total, 0.93; subscale, 0.84–0.92), CRPS [20] (total, 0.95; neuropathic subscale, 0.83), knee OA [22] (total, 0.88; subscale 0.75–0.81) and acute back pain [21] (total, 0.93; subscale, 0.77–0.84) patient populations. Inter-item correlations were also adequate. The adequate Cronbach’s alpha obtained signifies the absence of redundancy in the domains of SF-MPQ-2 thus confirming their unidimensionality [32] to capture the different pain characteristics they assess.

In the present study, ICC_2,1_ coefficients were good to excellent for all the SF-MPQ-2 domain scores (total, 0.93; subscales, 0.78–0.91), suggesting that they can adequately discriminate among patients at the individual level (total and continuous scale) and at the group level (all of the SF-MPQ-2 domains) [29, 54]. These results are comparable or better than previous findings reporting estimates among knee OA [22] (total scale, 0.90; subscales, 0.73–0.90) and mixed MSK patients [24, 55] (total scale, 0.90–0.94; subscales, 0.73–0.90). Although acceptable, the lower performance of the neuropathic subscale (0.78), with an ICC score that overlapped the ‘moderate’ confidence interval threshold (0.64–0.87), suggests greater variability on this subscale, which makes it more difficult to achieve a high ICC_2,1_ score.

Absolute reliability estimates allow clinicians to assess true change in a patient in comparison to change that might be expected from measurement error [30, 44]. Currently, no previous data have examined absolute reliability indices for the SF-MPQ-2 scores in any population. This makes direct interpretation and comparison difficult; however, our use of the Ostelo et al. [39] definition of SEM and MDC by percentages allows comparison across the domains of the SF-MPQ-2, and with its former version (SF-MPQ). The SEM for the total score (≤ 5% of total scale score) was ‘very good’ and comparable to that reported for the former version (SF-MPQ) among OA patients (≤ 3.64%) [56], but better than those seen among mixed MSK patients assessed with the Norwegian version of the SF-MPQ (≤ 10%) [41]. Although not as favorable as estimates noted on the total scale, the affective and intermittent/continuous subscales had ‘good’ SEM coefficients (< 10%), which were comparable to findings reported with the sensory subscale of the former SF-MPQ version among OA patients (< 10%) [56], and superior to that reported in a mixed MSK population (< 14%) [41]. Basically, SEM estimates for all the SF-MPQ-2 subscales were satisfactory and suggest an adequate evaluative capacity that can yield scores less prone to error when utilized by researchers/clinicians for MSK shoulder pain assessment over time.

The MDC scores represent the minimal change in scores after repeated administration that clinicians/researchers can interpret as not due to chance variation for an individual or group in a population [42]. The MDC_90indivdiual_ scores obtained for the SF-MPQ-2 domains implies that change at a magnitude equal or greater than 1.8 (neuropathic), 1.7 (affective), 1.8 (continuous), 2.3 (intermittent), 1.2 (total) points represents genuine improvement beyond chance with 90% confidence. The MDC scores for the total scale (≤ 12% of the total score of the scale) were comparable to previous studies with the former version (SF-MPQ) among OA patients (≤ 11.5%) and better than the results seen among mixed MSK patients (≤ 26.4% of total score). For the MDC_90group_ scores, the results obtained for the SF-MPQ-2 domains imply that a change of at least 0.4 (affective), 0.5 (intermittent), 0.3 (total), 0.4 (neuropathic), 0.4 (continuous) points must be observed in a group to be 90% confident that this was change beyond random or systematic error. In general, minimal detectable change scores are useful when interventions are administered; to be sure the intervention is effective, it must demonstrate change beyond the MDC score reported for the scale. Also, MDC_90group_ indices can be used for sample size estimation in a randomized controlled trial, as they determine the number of participants that will be needed to detect a change in the measure beyond error for a group, if the Minimal Clinically Important Difference (MCID) score for the population is unknown.

The Bland–Altman plots revealed satisfactory limits of agreement in support of the SF-MPQ-2 subscales. However, the interpretation of how far apart two measurements can be before they are no longer considered interchangeable depends on the contextual application [47]. The limits of agreement between the test–retest of the SF-MPQ-2 domains were reasonably smaller than those seen in previous studies of its former version (SF-MPQ) [41, 56], suggesting there is less variation between the test and the retest of the SF-MPQ-2 [50]. Furthermore, no bias was found in the measurements between the test–retest, as the inter-occasion mean difference was minimal. This suggests that learning or test accommodation are not issues with using the SF-MPQ-2; moreover, our compliance to recommended time intervals (3–7 days) [28, 29, 57] may have favored the agreement outcomes. The intermittent subscale had the greatest number of outliers of all the Bland–Altman plots (12%) and may be due to the volatile nature of the pain descriptors comprising the scale.

The SF-MPQ-2 total scores displayed the best reproducibility parameters in support of its relative, absolute and level of agreement parameters. This could be from the number of items contained in the scale. For instance, better ICC scores can be expected when variability is low. Variability decreases when a greater number of descriptors comprise a scale, in comparison to those with fewer descriptors [29]. As all 22 items of the SF-MPQ-2 contribute to the summary total scale scores, it is possible this favors reproducibility.

### Study limitations

While the present study findings provide preliminary evidence supporting the reproducibility of the SF-MPQ-2 for use in patients with shoulder disorders, it has several limitations. First, the study sample size (48 participants) was just under 50 participants which has been suggested as a benchmark by the COSMIN [58, 59]. However, in conflict with the COSMIN recommendation, our sample size calculation suggested at least 46 patients were required (see Appendix 1), which indicates our study was adequately powered. Second, the patient population were from a single tertiary referral practice and our findings may not be generalizable to a different context. Third, since participants completed the retest (Time 2) at home, we were unable to clarify instructions. However, independent completion is a requirement for routine administration. Further, the high level of agreement between scores of the tests and the absence of systematic bias suggest this was not a problem. Fourth, sample mean age was 62 (± 17) years, which may not adequately reflect the reliability of younger populations although shoulder pathology prevalence increases with age. Finally, we did not determine minimal clinically important difference.

## Conclusion

We conclude that the SF-MPQ-2 is satisfactorily internally consistent and provides good to excellent reproducibility coefficients (test–retest reliability and agreement) for multidimensional pain assessment among patients with musculoskeletal shoulder pain conditions. The total scale displays the best reproducibility coefficients. Additional research on the validity and responsiveness of the SF-MPQ-2 is still required in this population.

## Data Availability

This study data set is not assessible to the general public. However, the analysis of the dataset is assessible on reasonable request to the corresponding author, Samuel U. Jumbo.

## Appendix 1: Formula used for sample size calculation

$$\mathrm{n}=2+ \frac{0.5 k ({Z}_{\alpha }+ {Z}_{\beta }{)}^{2}}{{\delta }^{2}(k-1)}$$

where:

- k = number of occasions = 2
- Z_α_ represents the Z-value associated with the α value of interest. Therefore, Z-value (1-tailed) for 0.05 is equivalent to 1.645
- Z_β_ represents the Z-value associated with a Type II error. At power 80%, β equals 0.20, and Z-value (1-tailed) equals 0.842.
- δ = R value of Z transformed expected hypothesis minus R value of Z transformed null hypothesis, i.e. δ = Z _Rexpected_ – Z _Rnull_
  Where
  Z _Rexpected_ = reliability value expected from analysis
  Rexpected = 0.9
  Z _Rnull_ = lower confidence limit for the desired confidence interval width
- Z_Rexpected_ = $$0.5 \,natural\,\mathrm{ log}\frac{1+\left(k-1\right){R}_{expected}}{1-{R}_{expected}}$$  
- Z_Rexpected_ = $$0.5\, natural\,\mathrm{ log}\frac{1+\left(2-1\right)0.9}{1-0.9}$$ = 1.47
- Z_Rnull_ = $$0.5\, natural\,\mathrm{ log}\frac{1+\left(2-1\right){R}_{lowerlimit}}{1-{R}_{lowerlimit}}$$  
- R_lowerlimit_ = 0.09—0.10 = 0.80 (at 0.10 Confidence Interval width)
- Z _Rnull_ = $$0.5\, natural\,\mathrm{ log}\frac{1+\left(2-1\right)0.8}{1-0.8}$$ = 1.09
- δ = 1.47 – 1.09 = 0.38
- $$\mathrm{n }= 2+ \frac{0.5 k ({Z}_{\alpha }+ {Z}_{\beta }{)}^{2}}{{\delta }^{2}(k-1)} = 2+ \frac{0.5 (2) (1.645+0.842{)}^{2}}{{(0.38)}^{2}(2-1)}$$
- n = 46 patients

A sample size of **46** participants will be required for the reliability analysis.

## Abbreviations

SF-MPQ-2: Revised Short McGill Pain Questionnaire Version-2
SF-MPQ: Short-Form McGill Pain Questionnaire
ICC: Intraclass correlation
CI: Confidence interval
SEM: Standard error of measurement
MDC: Minimal detectable change
LoA: Limits of agreement
BA plot/analysis: Bland–Altman analysis/plots
COSMIN: COnsensus-based Standards for the selection of health Measurement INstruments
CPRS: Complex Regional Pain Syndrome
